# The Hemodynamic Trap: Fetal‐Type Posterior Cerebral Artery and Occult PICA Aneurysms

**DOI:** 10.1002/ccr3.73711

**Published:** 2026-10-14

**Authors:** Seyedeh Hamideh Khadem, Asal Ghari Saadati, Amirhossein Fathabadi, Farzaneh Khoroushi

**Affiliations:** ^1^ Department of Radiology, Faculty of Medicine Mashhad University of Medical Sciences Mashhad Iran; ^2^ Department of Medical Physics, Faculty of Medicine Mashhad University of Medical Sciences Mashhad Iran; ^3^ Medical Physics Research Center, Basic Sciences Research Institute Mashhad University of Medical Sciences Mashhad Iran

**Keywords:** computational hemodynamics, diagnostic pitfall, digital subtraction angiography, distal PICA aneurysm, fetal‐type posterior cerebral artery, isolated intraventricular hemorrhage, wall shear stress

## Abstract

Isolated intraventricular hemorrhage (IVH) is an uncommon presentation of ruptured aneurysms, often misdiagnosed as hypertensive hemorrhage. Distal posterior inferior cerebellar artery (PICA) aneurysms can easily be missed on computed tomography angiography (CTA) due to their small size and skull‐base artifacts. A 48‐year‐old female presented with a thunderclap headache. Initial imaging showed isolated IVH without subarachnoid hemorrhage. A primary CTA was read as negative, noting only a suspected plaque near the foramen magnum. However, multiformat reconstructions revealed a right‐sided fetal‐type posterior cerebral artery (fPCA) and a dominant left vertebral artery. High clinical suspicion prompted digital subtraction angiography (DSA), which confirmed a 3 × 3 mm distal PICA aneurysm exactly where the “plaque” was suspected on the CTA. This case highlights the limitations of CTA in the posterior fossa. It also suggests a hemodynamic mechanism for aneurysm formation: the combination of an fPCA and asymmetric vertebral inflow alters local blood flow. We hypothesize that this architectural mismatch may cause turbulence and increased wall shear stress, predisposing the distal PICA to aneurysm formation. In patients with isolated IVH and an fPCA variant, a negative CTA cannot definitively rule out a vascular lesion. Early DSA is essential to detect occult aneurysms and prevent subsequent rebleeding.

## Key Clinical Message

A negative CTA does not exclude a ruptured aneurysm in cases of isolated intraventricular hemorrhage; early digital subtraction angiography should be strongly considered. Additionally, the triad of isolated IVH, fetal‐type posterior cerebral artery, and vertebrobasilar asymmetry may indicate a hemodynamically unstable environment predisposing patients to distal PICA aneurysms.

## Graphical abstract

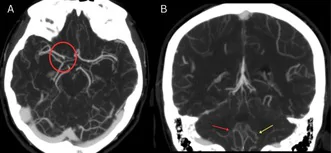

## 1 Introduction

The posterior inferior cerebellar artery (PICA) exhibits profound anatomical complexity as it perfuses the medulla and the inferior cerebellum. Aneurysms originating from its distal segments are exceptionally rare clinical entities, comprising approximately 0.5%–3.0% of all intracranial aneurysms [1, 2]. Rupture of these aneurysms predominantly presents as classical subarachnoid hemorrhage (SAH); however, due to their intimate anatomical proximity to the fourth ventricle and the foramina of Luschka, aneurysmal rupture can occasionally precipitate isolated intraventricular hemorrhage (IVH) via a direct arterial jetting phenomenon [3, 4]. This atypical presentation is frequently misattributed in the emergency setting to hypertensive microangiopathy or occult coagulopathies, fostering a false sense of diagnostic security and delaying critical endovascular interventions [5, 6].

Computed tomography angiography (CTA) is widely regarded as the first‐line non‐invasive modality for intracranial vascular assessment. However, its diagnostic performance declines in the posterior fossa, where the high attenuation of the petrous temporal and occipital bones produces beam‐hardening and photon‐starvation artifacts that degrade vessel conspicuity and obscure small aneurysms [7, 8]. Furthermore, the limited spatial resolution of standard clinical CTA—typically bounded by voxel sizes of 0.5–0.6 mm—renders sub‐centimeter, tortuous aneurysms highly susceptible to partial‐volume averaging [8]. Consequently, small saccular aneurysms may be mischaracterized as atherosclerotic plaques or benign vascular loops on CTA, contributing to false‐negative interpretations that delay definitive digital subtraction angiography (DSA) [9, 10].

Beyond structural imaging limits, anatomical variants of the Circle of Willis influence the biomechanical microenvironment of the cerebral vasculature. The fetal‐type posterior cerebral artery (fPCA) variant redirects flow from the internal carotid artery to the PCA, reducing basilar artery contribution to posterior circulation flow. This creates hemodynamic imbalances that can promote vascular pathology [11]. From a computational fluid dynamics (CFD) perspective, fPCA variants—particularly when combined with asymmetric vertebral artery dominance—alter basilar flow dynamics, reducing laminar shear patterns and elevating oscillatory shear stress in posterior circulation bifurcation zones [11, 12]. Such geometric discordance may precipitate secondary flow currents, focal turbulence, and pathologically amplified wall shear stress (WSS). Prolonged exposure to these aberrant shear forces is hypothesized to act as a catalyst for endothelial degradation, concluding in saccular aneurysm formation at distal bifurcations [12, 13].

Herein, we report an exceptionally deceptive case of isolated IVH originating from a diminutive, ruptured distal PICA aneurysm that entirely evaded initial CTA detection. The concurrent identification of a unilateral fPCA and profound vertebrobasilar asymmetry in this patient underscores a compelling hemodynamic hypothesis for aneurysm development, highlighting the critical intersection of anatomical variance, diagnostic imaging limitations, and vascular biophysics.

## 2 Case History

A 48‐year‐old previously healthy female presented to the emergency department with a sudden‐onset, explosive thunderclap headache (visual analog scale 10/10), accompanied by intractable emesis and profuse diaphoresis. Collateral history indicated a brief, transient episode of mild disorientation immediately following the headache onset, which had completely resolved by the time of neurosurgical assessment. The patient had no documented history of systemic hypertension, diabetes mellitus, coagulopathy, or familial connective tissue disorders. Upon physical examination, she was fully alert, hemodynamically stable, and exhibited no focal sensorimotor deficits or cranial neuropathies (Glasgow Coma Scale: 15). Classical signs of meningeal irritation, such as nuchal rigidity, were notably absent. Her clinical severity was stratified as Hunt and Hess Grade II and World Federation of Neurosurgical Societies (WFNS) Grade I.

## 3 Differential Diagnosis, Investigations and Treatment

Emergent non‐contrast computed tomography (NCCT) of the brain revealed acute primary IVH. Prominent hyperdense sedimentation of blood products was observed layering within the dependent occipital horns of both lateral ventricles (Figure 1). Conspicuously, there was a profound absence of diffuse cisternal or cortical SAH. This isolated IVH pattern is highly atypical for standard aneurysmal rupture and strongly implicates a paraventricular source, such as a distal PICA aneurysm discharging retrogradely through the foramina of Luschka or Magendie directly into the ventricular system, effectively bypassing the subarachnoid space.

**FIGURE 1:**
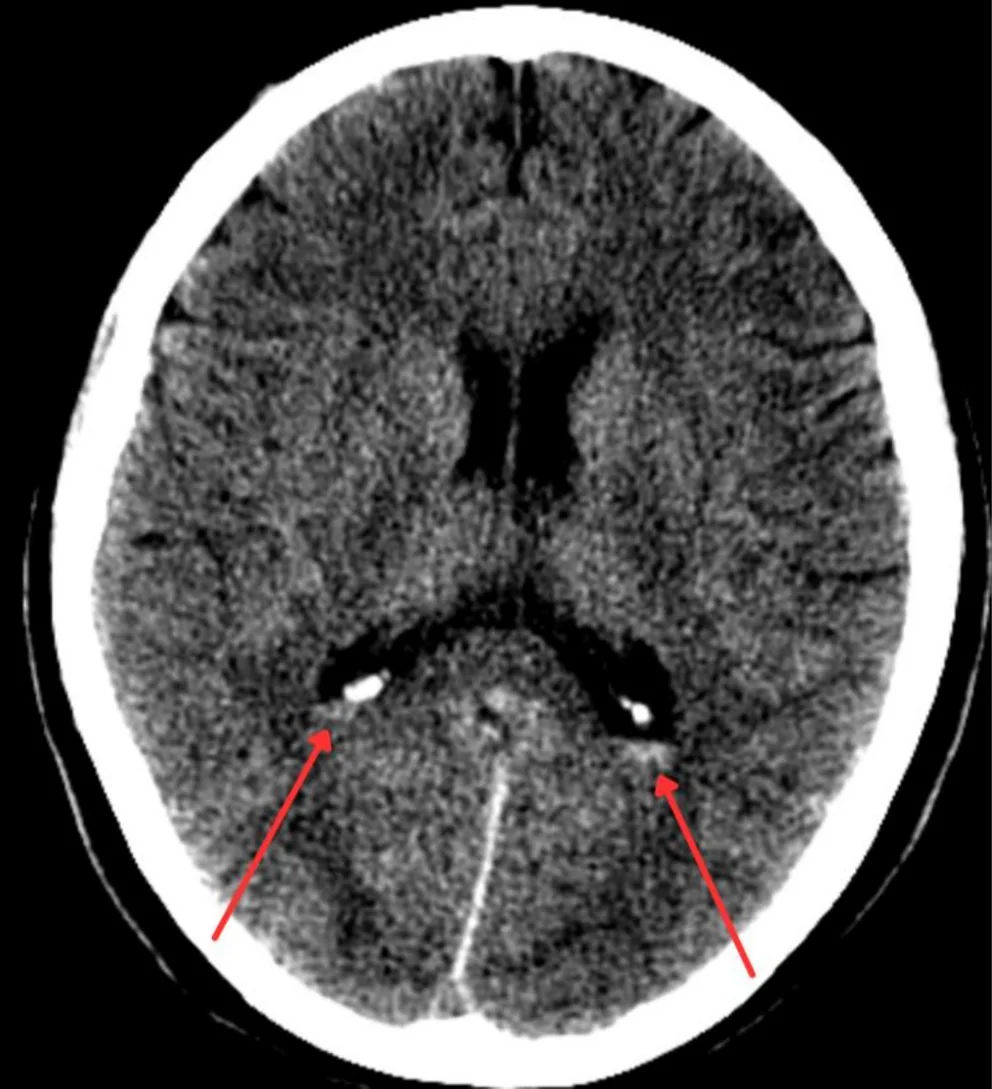
Non‐contrast computed tomography (NCCT) demonstrating acute primary intraventricular hemorrhage. Red arrows indicate the hyperdense sedimentation of blood products layering within the dependent occipital horns of the lateral ventricles, with a conspicuous absence of diffuse cisternal subarachnoid hemorrhage.

Subsequent multidetector CTA of the head and neck was formally interpreted as negative for macroscopic intracranial aneurysms. The initial radiological report documented only a subtle, non‐calcified hypodensity near the left foramen magnum, which was misclassified as an atherosclerotic plaque or a complex overlapping vascular loop (Figure 2A). From an imaging physics perspective, this represents a classical false‐negative pitfall: sub‐centimeter lesions in the posterior fossa are frequently obscured by severe photon starvation and beam‐hardening artifacts emanating from the dense temporal and occipital bones. Furthermore, a diminutive 3 mm aneurysm is highly vulnerable to partial‐volume averaging, causing the lesion to visually mimic a plaque.

**FIGURE 2:**
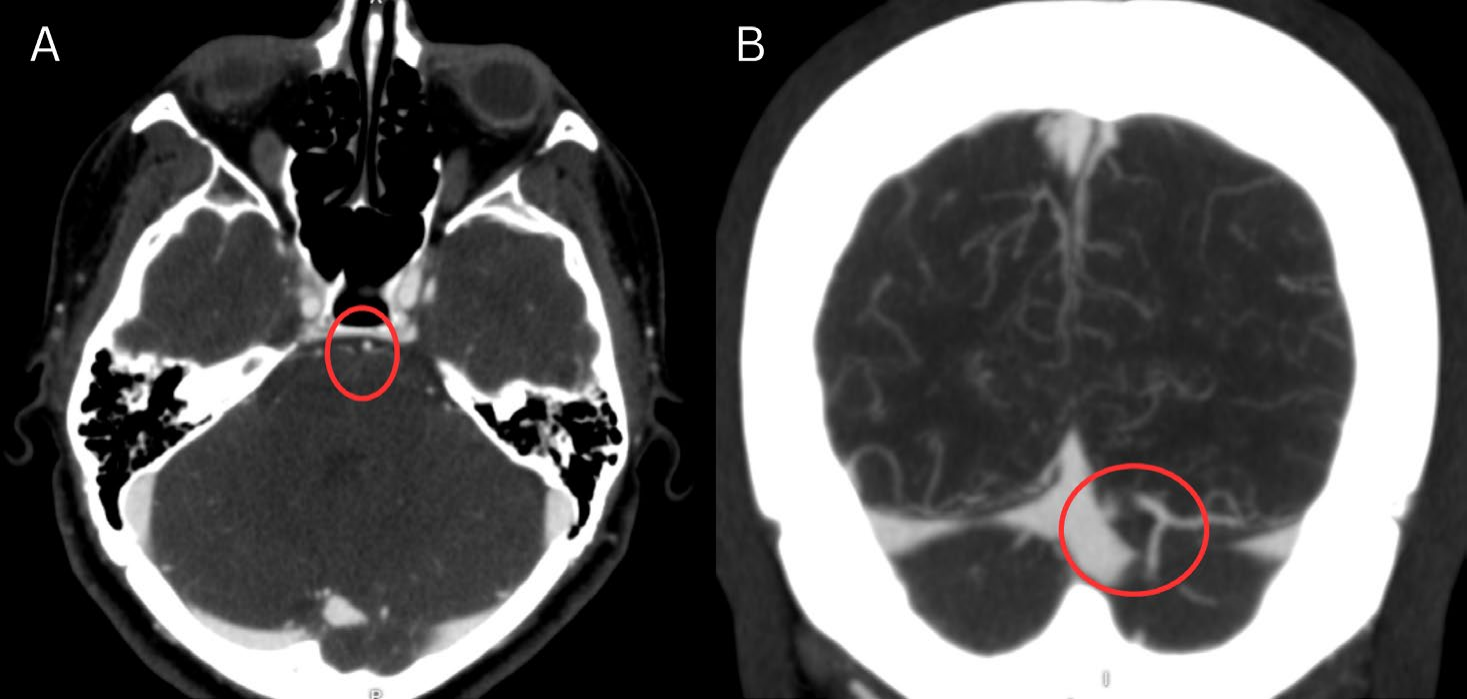
Diagnostic limitations of standard axial computed tomography angiography (CTA) in the posterior fossa. (A) Axial source image showing a subtle, 3 mm structure near the brainstem (red circle), initially misinterpreted as a hypodense plaque due to severe beam‐hardening and partial‐volume artifacts. (B) Coronal Maximum Intensity Projection (MIP) reconstruction of the same CTA dataset. Retrospective analysis clearly reveals the true nature of the lesion as a saccular outpouching (red circle) arising from the left PICA. This highlights the necessity of multiplanar reconstructions to overcome spatial resolution limits in artifact‐prone regions.

Crucially, retrospective multiformat Maximum Intensity Projection (MIP) reconstructions illuminated a variant cerebrovascular architecture. Axial MIPs confirmed a unilateral right‐sided fPCA, characterized by an absent P1 segment (Figure 3A). Concurrently, coronal MIPs demonstrated a striking inflow asymmetry: a dominant, highly tortuous left vertebral artery paired with a hypoplastic right vertebral artery (Figure 3B). This geometrical configuration may alter physiological basilar outflow. Consequently, the high‐velocity, dominant left vertebral inflow could generate secondary flow patterns along the ipsilateral PICA.

**FIGURE 3:**
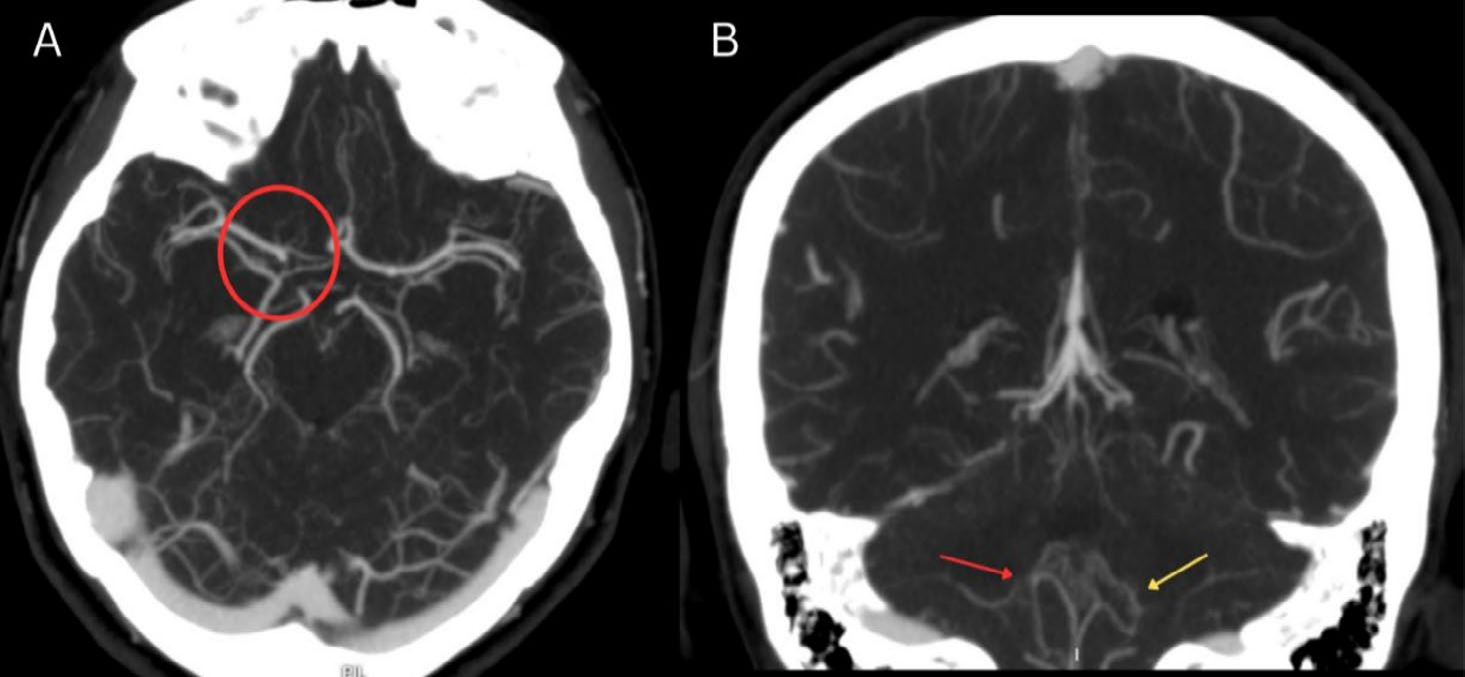
Hemodynamic and structural asymmetry of the vertebrobasilar system. (A) Axial Maximum Intensity Projection (MIP) demonstrating a right‐sided fetal‐type posterior cerebral artery (fPCA). The right PCA arises directly from the internal carotid artery (red circle), with an absent P1 segment. (B) Coronal MIP reveals striking inflow asymmetry, characterized by a dominant, tortuous left vertebral artery (yellow arrow) and a hypoplastic right vertebral artery (red arrow). This combination of altered outflow (fPCA) and asymmetric inflow likely induces extreme wall shear stress and local turbulence at the left distal PICA, biomechanically predisposing it to aneurysm formation.

Given the unexplained isolated IVH, a known red flag for occult ventricular or paraventricular vascular lesions, and the equivocal CTA findings in an artifact‐prone region, the multidisciplinary neurovascular board mandated a four‐vessel DSA within 24 h of admission. Selective micro‐catheterization and 3D rotational angiography of the left vertebral artery definitively unmasked a 3 × 3 mm saccular aneurysm. The lesion originated directly from the highly tortuous telovelotonsillar segment of the left PICA (Figure 4). Retrospective spatial coregistration confirmed that this actively ruptured dome occupied the exact geometric coordinates of the plaque misidentified on the initial CTA. Following definitive diagnosis, the patient underwent targeted endovascular coil embolization using detachable coils to secure the dome and neutralize the risk of re‐rupture. The aneurysm was successfully secured. Follow‐up imaging at 3 months demonstrated complete occlusion of the aneurysm. Clinically, the patient made an uneventful recovery with a modified Rankin Scale (mRS) score of 0 upon discharge.

**FIGURE 4:**
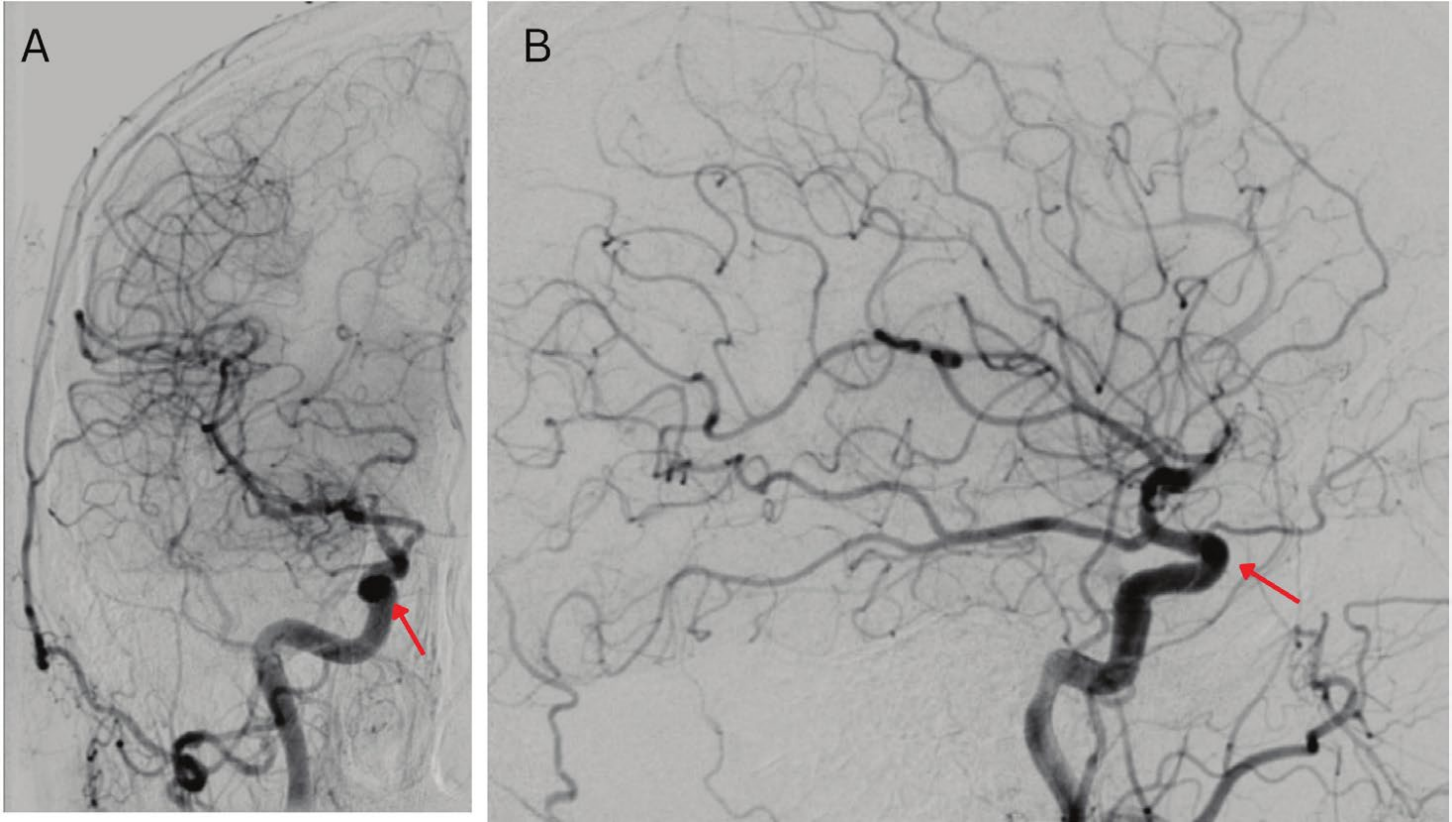
Digital Subtraction Angiography (DSA) confirming the vascular pathology. (A) Anteroposterior (AP) and (B) lateral projections of the left vertebral artery injection. The red arrows precisely identify a 3 mm saccular aneurysm arising from the proximal segment of the left posterior inferior cerebellar artery (PICA), corresponding to the exact spatial coordinates of the misinterpreted lesion on the initial CTA.

## 4 Conclusion and Results

Distal PICA aneurysms can mimic hypertensive IVH, and these diminutive lesions are frequently obscured on standard CTA by skull‐base artifacts. While our proposed mechanism remains a hypothesis requiring future CFD validation, the specific anatomical configuration of an fPCA and vertebrobasilar asymmetry may create a hemodynamically unstable environment that may mechanically promote aneurysm formation. Given the physical limitations of non‐invasive imaging in the posterior fossa, emergent DSA is strongly recommended for diagnosing occult vascular lesions in these high‐risk scenarios to avert subsequent rebleeding.

## 5 Discussion

Primary isolated IVH—without parenchymal hematoma or diffuse SAH—is rare, comprising approximately 1%–3% of spontaneous intracranial hemorrhages. It is most commonly attributed to hypertensive microangiopathy, arteriovenous malformations (AVMs), aneurysms, or choroid plexus lesions [6, 14]. Aneurysms of the distal posterior inferior cerebellar artery (PICA) are exceedingly rare culprits. However, because the PICA courses intimately along the roof of the fourth ventricle, a ruptured aneurysm in this territory can force arterial blood directly into the ventricular system, bypassing the subarachnoid basal cisterns [1, 2]. This atypical presentation frequently delays mandatory neurovascular interventions, escalating the risk of re‐rupture [1, 15]. While previous reports have documented isolated IVH secondary to distal PICA aneurysms [3, 4], the present case is distinct. To our knowledge, this is the first report to describe the concurrent triad of isolated IVH, an fPCA, and vertebrobasilar asymmetry, suggesting a novel morphological predisposition.

As previously noted, the diagnostic failure of the initial CTA underscores the inherent physical limitations such as beam‐hardening and partial‐volume artifacts when imaging sub‐centimeter lesions in the posterior fossa [7, 8, 10]. This physical pitfall necessitates a low threshold for DSA when clinical suspicion persists.

A compelling aspect of this case is the potential biomechanical and hemodynamic pathogenesis of the aneurysm, dictated by the patient's underlying vascular architecture. The concurrent identification of a fPCA and a dominant, highly tortuous left vertebral artery creates an asymmetric fluid dynamics microenvironment. Physiologically, the basilar artery acts as a primary sink for vertebrobasilar inflow. The presence of a unilateral fPCA fundamentally curtails this downstream basilar outflow demand [11, 16]. When this truncated distal resistance is coupled with high‐velocity, asymmetric inflow from a dominant vertebral artery, the resulting hemodynamic mismatch disrupts laminar flow.

While no patient‐specific CFD modeling was performed for this case, we infer from established biomechanical principles that this specific vascular configuration may direct high‐momentum inflow to impinge on the branching origin of the PICA. This architectural discordance may promote secondary flow separation, local turbulence, and elevated WSS at the distal branch point [12, 17]. Over time, prolonged exposure to elevated WSS is a well‐established driver of endothelial degradation and saccular aneurysm formation. The precise spatial colocalization of the ruptured aneurysm on the distal loop of the dominant vertebral system in our patient strongly supports this inferential hypothesis.

## Author Contributions

**Seyedeh Hamideh Khadem:** conceptualization, investigation, visualization, writing – original draft. **Asal Ghari Saadati:** data curation, formal analysis, writing – review and editing. **Amirhossein Fathabadi:** formal analysis, methodology, writing – review and editing. **Farzaneh Khoroushi:** conceptualization, project administration, supervision, validation, writing – review and editing.

## Funding

The authors have nothing to report.

## Consent

Written informed consent was obtained from the patient for the publication of this case report and any accompanying images.

## Conflicts of Interest

The authors declare no conflicts of interest.

## Data Availability

The supporting data are available from the corresponding author upon reasonable request.

## References

[1] H. L. Miao , D. Y. Zhang , T. Wang , X. T. Jiao , and L. Q. Jiao , “Clinical Importance of the Posterior Inferior Cerebellar Artery: A Review of the Literature,” International Journal of Medical Sciences 17, no. 18 (2020): 3005–3019. 10.7150/ijms.49137 PMC7646108 33173421

[2] J. Zhu , L. Yin , Y. Che , et al., “Treatment of a Giant Complicated Distal Posterior Inferior Cerebellar Artery Aneurysm: A Case Report and Literature Review,” Experimental and Therapeutic Medicine 20, no. 1 (2020): 395–400. 10.3892/etm.2020.8724 PMC7281963 32537003

[3] D. F. Kallmes , G. Lanzino , J. E. Dix , et al., “Patterns of Hemorrhage With Ruptured Posterior Inferior Cerebellar Artery Aneurysms: CT Findings in 44 Cases,” American Journal of Roentgenology 169, no. 4 (1997): 1169–1171. 10.2214/ajr.169.4.9308484 9308484

[4] A. Savardekar , M. K. Tewari , R. Garg , V. Gupta , and C. Ahuja , “Extracranially Located PICA Aneurysm Presenting With Supratentorial IVH: A Rare Event With Diagnostic Pitfalls,” Journal of Neurosciences in Rural Practice 4, no. Suppl 1 (2013): S99–S101. 10.4103/0976-3147.116430 PMC3808075 24174813

[5] I. Barnaure , A. C. Liberato , R. G. Gonzalez , and J. M. Romero , “Isolated Intraventricular Haemorrhage in Adults,” British Journal of Radiology 90, no. 1069 (2017): 20160779. 10.1259/bjr.20160779 PMC5605039 27805421

[6] A. S. Das , R. W. Regenhardt , E. Gokcal , et al., “Idiopathic Primary Intraventricular Hemorrhage and Cerebral Small Vessel Disease,” International Journal of Stroke 17, no. 6 (2022): 645–653. 10.1177/17474930211043957 PMC10947797 34427471

[7] A. Guarnizo , K. Farah , D. A. Lelli , D. Tse , and N. Zakhari , “Limited Usefulness of Routine Head and Neck CT Angiogram in the Imaging Assessment of Dizziness in the Emergency Department,” Neuroradiology Journal 34, no. 4 (2021): 335–340. 10.1177/1971400920988665 PMC8447815 33487089

[8] C. J. van Asch , B. K. Velthuis , G. J. Rinkel , et al., “Diagnostic Yield and Accuracy of CT Angiography, MR Angiography, and Digital Subtraction Angiography for Detection of Macrovascular Causes of Intracerebral Haemorrhage: Prospective, Multicentre Cohort Study,” BMJ 351 (2015): h5762. 10.1136/bmj.h5762 PMC4637845 26553142

[9] S.‐H. Ng , H.‐F. Wong , S.‐F. Ko , et al., “CT Angiography of Intracranial Aneurysms: Advantages and Pitfalls,” European Journal of Radiology 25, no. 1 (1997): 14–19. 10.1016/s0720-048x(97)01160-1 9248792

[10] A. R. Hochberg , R. Rojas , A. J. Thomas , A. S. Reddy , and R. A. Bhadelia , “Accuracy of On‐Call Resident Interpretation of CT Angiography for Intracranial Aneurysm in Subarachnoid Hemorrhage,” American Journal of Roentgenology 197, no. 6 (2011): 1436–1441. 10.2214/AJR.11.6782 22109300

[11] L. Zheng , Y. Chen , X. Lin , et al., “Relationship Between Fetal‐Type Posterior Cerebral Artery and Basilar Artery Atherosclerosis,” Frontiers in Neurology 16 (2025): 1533281. 10.3389/fneur.2025.1533281 PMC11983884 40212612

[12] T. T. G. Le , S. W. Ryu , J. J. Yoon , T. Nam , and J. Ryu , “Assessing the Impact of Fetal‐Type Posterior Cerebral Artery Variations on Cerebral Hemodynamics,” Physics of Fluids 36, no. 10 (2024): 101901.

[13] R. Schwab , R. Janiszewski , E. Fuchs , et al., “Fetal‐Type Posterior Communicating Artery Increases Hemodynamic Stress in Posterior Communicating Artery Bifurcation Aneurysms: A CFD‐Based Analysis,” Neuroradiology 67, no. 9 (2025): 2471–2481. 10.1007/s00234-025-03785-w PMC12546534 40974399

[14] A. Pai , A. Hegde , R. Nair , and G. Menon , “Adult Primary Intraventricular Hemorrhage: Clinical Characteristics and Outcomes,” Journal of Neurosciences in Rural Practice 11, no. 4 (2020): 623–628. 10.1055/s-0040-1716770 PMC7595788 33144801

[15] H. Lantigua , S. Ortega‐Gutierrez , J. M. Schmidt , et al., “Subarachnoid Hemorrhage: Who Dies, and Why?,” Critical Care 19, no. 1 (2015): 309. 10.1186/s13054-015-1036-0 PMC4556224 26330064

[16] G. S. Park and J. S. Park , “Anatomical Differences of the Vertebrobasilar Artery Between Normal Subjects and Patients With Cerebral Infarction,” Medicine (Baltimore) 103, no. 32 (2024): e39105. 10.1097/MD.0000000000039105 PMC11315531 39121322

[17] A. K. Wake‐Buck , J. C. Gatenby , and J. C. Gore , “Hemodynamic Characteristics of the Vertebrobasilar System Analyzed Using MRI‐Based Models,” PLoS One 7, no. 12 (2012): e51346. 10.1371/journal.pone.0051346 PMC3519605 23251503

